# Peripheral arterial pathology and osteoarthritis of the knee: US examination of arterial wall stiffness, thickness, and flow characteristics

**DOI:** 10.1016/j.ocarto.2024.100537

**Published:** 2024-10-29

**Authors:** Jon Olansen, Minglang Yin, Janine Molino, Thomas Carruthers, Derek Jenkins, George Karniadakis, Roy K. Aaron

**Affiliations:** a Department of Orthopedic Surgery, Warren Alpert Medical School of Brown University, Providence, RI 02903, USA; b Department of Biomedical Engineering, Johns Hopkins University, Baltimore, MD 21287, USA; c Division of Vascular Surgery, Department of Surgery, Warren Alpert Medical School of Brown University, Providence, RI, USA; d Division of Applied Mathematics, Brown University, Providence, RI 02912, USA

**Keywords:** Vascular disease, Osteoarthritis, Pulse wave velocity, Arterial stiffness, Endothelin

## Abstract

**Background:**

Osteoarthritis (OA) is a widespread chronic joint disorder characterized by the degeneration of articular cartilage, extensive bone remodeling, ligamentous fibrosis, and synovial inflammation impacting millions. Shared factors like phenotypic similarities, hypofibrinolysis, and inflammation constitute similarities in pathophysiology and clinical manifestations between OA and peripheral vascular disease (PVD). This study investigated peripheral arterial flow characteristics, vascular wall thickness, and stiffness in knee OA to clarify a potential association with early atherosclerosis.

**Methods:**

The OA cohort consisted of 35 knees with a mean age of 69 years. The control cohort consisted of 58 knees with a mean age of 68 years. Subjects underwent arterial ultrasound scanning of the common femoral, superficial femoral, and popliteal arteries. Data measured included peak systolic volumetric flow, intima-media thickness, systolic and diastolic vessel diameter, and simultaneous EKG and flow curves. Structural and functional vascular data were quantified using the incremental Young's modulus, pulse wave velocity (PWV), and distensibility.

**Results:**

Significantly elevated arterial volumetric flow, measures of arterial stiffness, and intima-media wall thickness were observed in those with OA compared to those without. PWV as calculated by the Bramwell-Hill equation were found to be significantly greater in all three vessels of patients with OA.

**Conclusions:**

This study supports the association between peripheral arterial pathology and knee OA, consistent with shared clinical and phenotypic traits. The observed characteristics of early vascular pathology suggest potential pathophysiologic linkages between OA and PVD. This foundational framework provides avenues for mechanistic studies exploring the relationship between these two disease processes.

## 1 Introduction

Patients with osteoarthritis (OA) exhibit a higher than expected clinical incidence of cardiovascular comorbidities including ischemic coronary disease, cerebrovascular and peripheral arterial disease, and venous thromboembolism [[1], [2], [3], [4]]. Hip OA has been shown to be associated with a 25% increase in cardiovascular mortality [5]. Another study also reported a higher risk of ischemic heart disease in individuals with OA [6]. Two other reviews supported the observation of association between OA and peripheral vascular disease (PVD). A meta-analysis of 15 studies involving 80,911 OA patients confirmed a 24% increased risk of PVD in OA patients versus the general population (RR: 1.24, *P* ​< ​0.001) [7] A systematic review showed a relative prevalence of PVD of 38% in subjects with OA as compared to 9% in control subjects (*P* ​= ​0.01) [8]. Both OA and cardiovascular diseases share phenotypic characteristics, including obesity, hypertension, insulin insensitivity, hypercoagulation, and circulating inflammatory proteins suggesting potentially important shared pathophysiological relationships [3].

Several studies have reported isolated vascular pathologies in several forms of OA [1,3]. Structural and physiologic abnormalities have been demonstrated in retinal arterioles, and in carotid and popliteal arteries, in patients with generalized and hand OA [9,10]. A positive association has been reported between popliteal artery wall thickness and OA [11]. Impaired aortic elastic properties have been found in individuals with late-stage OA [12]. Other studies have presented a less convincing association with considerable variability among arteries in both normal and OA patients [13,14]. Technical precision of some devices did not meet the Artery Society accuracy criteria [14,15]. Despite the demonstration of isolated vascular abnormalities in certain types of OA, convincing evidence of comprehensive arterial pathology is still elusive for both sexes and in hip and knee OA [9,13]. Further, the significance of the shared clinical, phenotypic, and physiologic features between PVD and OA is not known and deserves more investigation. While the observations to date are provocative, they are incomplete and a thorough assessment of arterial abnormalities in OA is lacking and is addressed in this study.

Abnormalities of the arterial wall and related alterations in blood flow have been associated with systemic cardiac and vascular disease. Arterial wall stiffness is an established indicator of cardiovascular risk [16]. One study, a meta-analysis of 17 longitudinal studies involving 15,877 patients followed for a mean of 7.7 years reported a pooled relative risk of cardiovascular disease of 2.26 (95% Confidence Interval: 1.89–2.70, in 14/17 studies) in patients with increased arterial stiffness compared to normal populations [17]. Arterial stiffness has been associated particularly with increased pulse pressure, left ventricular hypertrophy, and subendocardial ischemia [18]. Increased intima-media wall thickness (IMT) has also been related to a greater incidence of clinical cardiovascular events and risk factors for vascular disease [19].

This study was undertaken to explore the relationships between lower extremity arterial pathology and OA of the knee with the goal of a detailed characterization of smaller arterial physiology in patients without clinically recognizable vascular disease. Using ultrasound, the study examined arteries ipsilateral to knee OA for volumetric blood flow, pulse wave velocity (PWV), and vessel wall structure and mechanics, and compares them to the same observations in normal knees. The hypothesis of this study was that abnormal limb arterial wall stiffness and abnormal blood flow, indicative of cardiovascular pathology, coexist with knee OA to a significantly greater degree than in subjects without knee OA, suggesting potential pathophysiologic relationships between OA and cardiovascular disease. Possibilities that OA and PVD share common vascular characteristics may provide a physiologic platform on which to link the mechanisms of early PVD with OA, and from which to base mechanistic studies and provide insights into clinically useful physiological markers and disease modifying therapies.

## 2 Methods

The study was approved by the IRB of the Lifespan Academic Medical Center. Written informed consent was obtained from all subjects involved in the research study. Research subjects, recruited from The Miriam Hospital joint replacement center and from the community, consisted of 58 asymptomatic knees (controls) and 35 symptomatic knees with known OA.

### 2.1 Clinical assessment

All research subjects were screened for a history of symptomatic cardiovascular disease, including hypertension, diabetes, hyperlipidemia, MI, arrhythmias, thromboembolic events, or any other arterial and venous events and interventions, and any knees with a positive cardiovascular history were excluded. All limbs were examined for edema, presence of pedal pulses, dependent rubor, as well as an appropriate capillary refill and ankle-brachial index. Abnormal ankle-brachial index has been shown to be a reliable measurement of the presence of peripheral artery disease in the microvasculature [20]. Eligibility criteria included a normal vascular examination with a normal ankle-brachial index of 0.9–1.4, capillary refill <3 ​s, and oxygen saturation of >94%. No knees entered into the study had clinically manifest symptoms of cardiovascular disease. All knees underwent a standard orthopedic history and examination for gait, tenderness and pain, synovitis, effusion, alignment, range of motion, stiffness, stability, crepitation, and strength. All knees were examined radiographically with standing antero-posterior, semi-flexed, and lateral views, and sitting sunrise views. Radiographs were evaluated by an experienced arthritis surgeon with extensive experience reading knee films. The radiographic measurements were only used to qualitatively categorize subjects by the presence or absence of OA as per our eligibility criteria. Measurements of the joint space width in the medial, or most affected, compartment were made to qualitatively categorize subjects by the presence or absence of knee OA as per the eligibility criteria. Knees were qualitatively graded on the Kellgren-Lawrence scale. Because of reported difficulties distinguishing K-L grades 1 and 2, only knees with grades 3 and 4 were included as OA of the knee [21].

Knees included in the normal (control) cohort were asymptomatic and had normal knee function, a normal knee examination, and normal X-rays. All knees included in the OA group were symptomatic and many were considering or scheduled for TKR. Symptoms consisted primarily of pain, instability, and/or weakness. Knee examination revealed one or several of the manifestations of OA prominently, synovial pain, crepitation, instability, and/or angulatory deformity. Radiographs were scored as K-L 3 or 4 with no ambiguity as to the characteristics of OA including subchondral plate sclerosis, osteophytes, joint space narrowing, and abnormal tibiofemoral angle.

### 2.2 Vascular evaluation

**Fig. 1:**
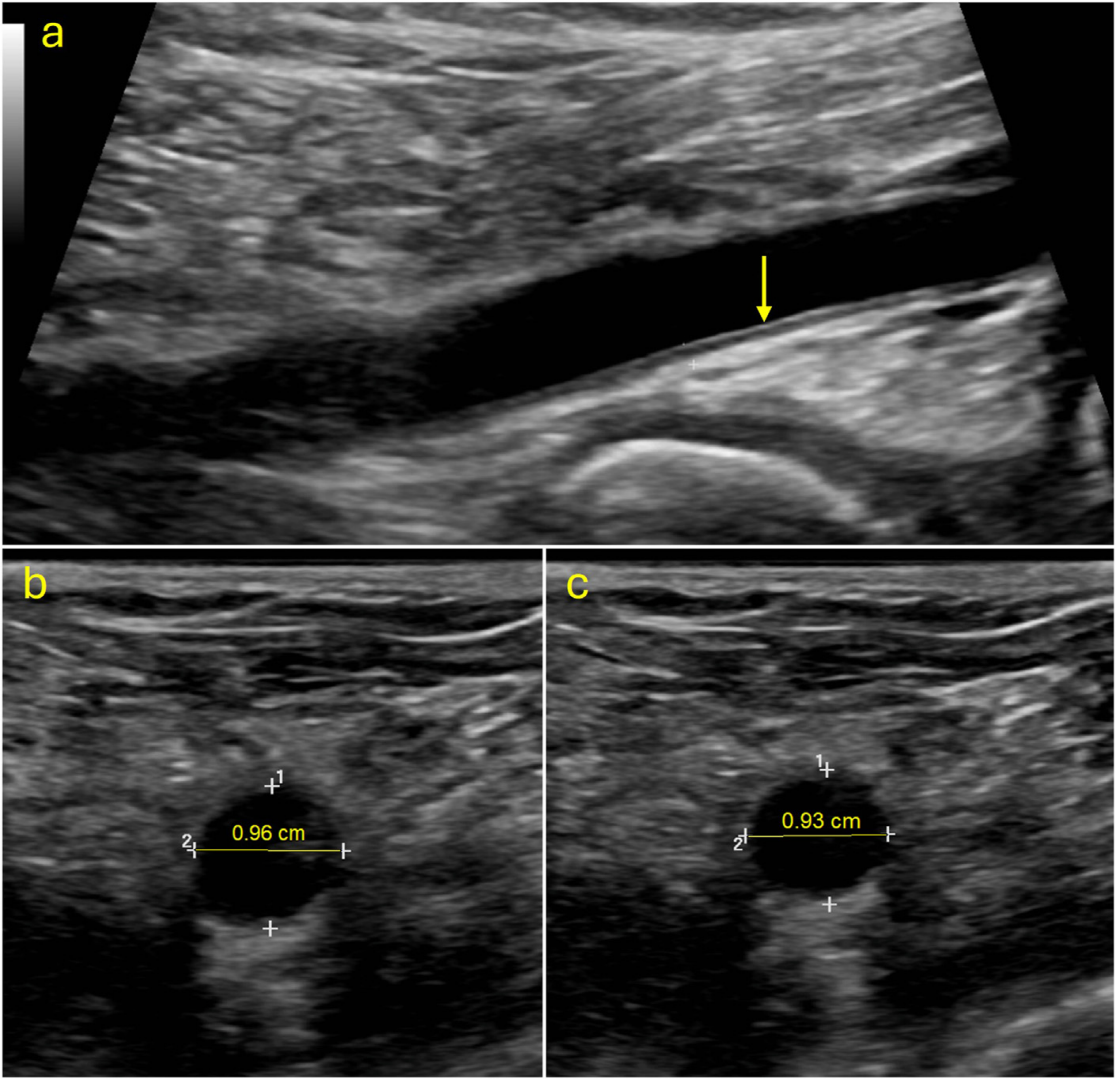
**Sonographic images of structural vessel properties captured using the GE Logiq9 system**. (a) Intima-media thickness of the popliteal artery (arrow). (b) Systolic cross-section of the common femoral. (c) Diastolic cross-section of the CFA. Area change was measured using the mean cross-sectional diameter differences between systole and diastole.

Noninvasive Doppler color duplex ultrasound was performed in all limbs with a GE Logiq 9 ultrasound instrument with a 9 ​MHz linear transducer [22]. Simultaneous EKG recordings were made and displayed on the same time scale as the arterial pulse waves. Three vessels, the common femoral (CFA), superficial femoral (SFA), and popliteal (Pop) arteries, were chosen for evaluation due to their clinical relevance, their ability to be reliably appreciated on ultrasound, and their direct implication in the development of lower extremity atherosclerosis [23]. Smaller vessels were not measured due to the understanding that increased, upstream vessel stiffness leads to excessive penetration of pulsatility in small vessels and, therefore, similar changes to shear stresses [24,25]. For arthritic knees, measurements were made ipsilateral to the arthritic knee. Several measurements and calculations were made to search for attributes of early arterial wall disease. Volumetric flow (ml/min) was measured in the three vessels. (IMT) was measured with B-mode duplex scanning and was defined by two parallel echogenic lines corresponding to the lumen-intima and media-adventitia interfaces (Fig. 1a). Systolic and diastolic luminal areas were measured in the cross-sectional plane and the area change with the cardiac cycle was calculated (Fig. 1b and c) [26].

**Fig. 2:**
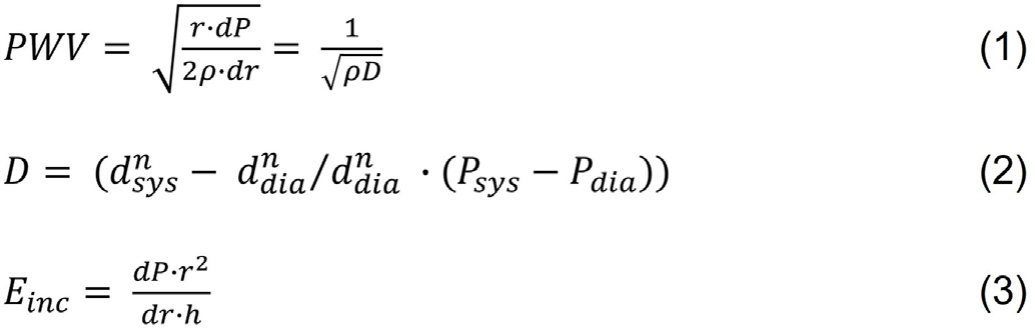
**Three equations used to calculate flow, structural properties, and material properties in the limb vessels.** (1) Bramwell-Hill equation used to calculate PWV where r ​= ​radius, dP ​= ​pressure difference between systole and diastole, ρ ​= ​blood density, D ​= ​distensibility. (2) Distensibility equation, d ​= ​diameter, P ​= ​pressure [23]. (3) Incremental Young's Modulus (E_inc_) equation, h ​= ​wall thickness, dP ​= ​pressure difference between systole and diastole, r ​= ​radius, dr ​= ​radius difference between systole and diastole [25].

Arterial stiffness is reflected by various descriptors of the vascular wall response to pulsatile blood flow and can be described as structural or material. Several metrics have been used to quantify structural arterial stiffness and their formulas for calculation are seen in Fig. 2 [27]. Compliance is defined by the change in luminal area per unit pressure and is expressed by vessel distensibility. Young's modulus, a metric to describe the mechanical stiffness of a material defined as deformation in response to stress, is used here to describe material stiffness. However, biological tissue almost always exhibits nonlinearity regarding its stress-strain relationship, and therefore it cannot be simply characterized by a single Young's modulus [28]. Instead, the incremental Young's modulus (E_inc_) is a better descriptor of the local slope of the stress-strain curve *in vivo* and was used as a stiffness-related metric to test our hypothesis [29]. Greater vessel stiffness corresponds to a larger incremental Young's modulus.

**Fig. 3:**
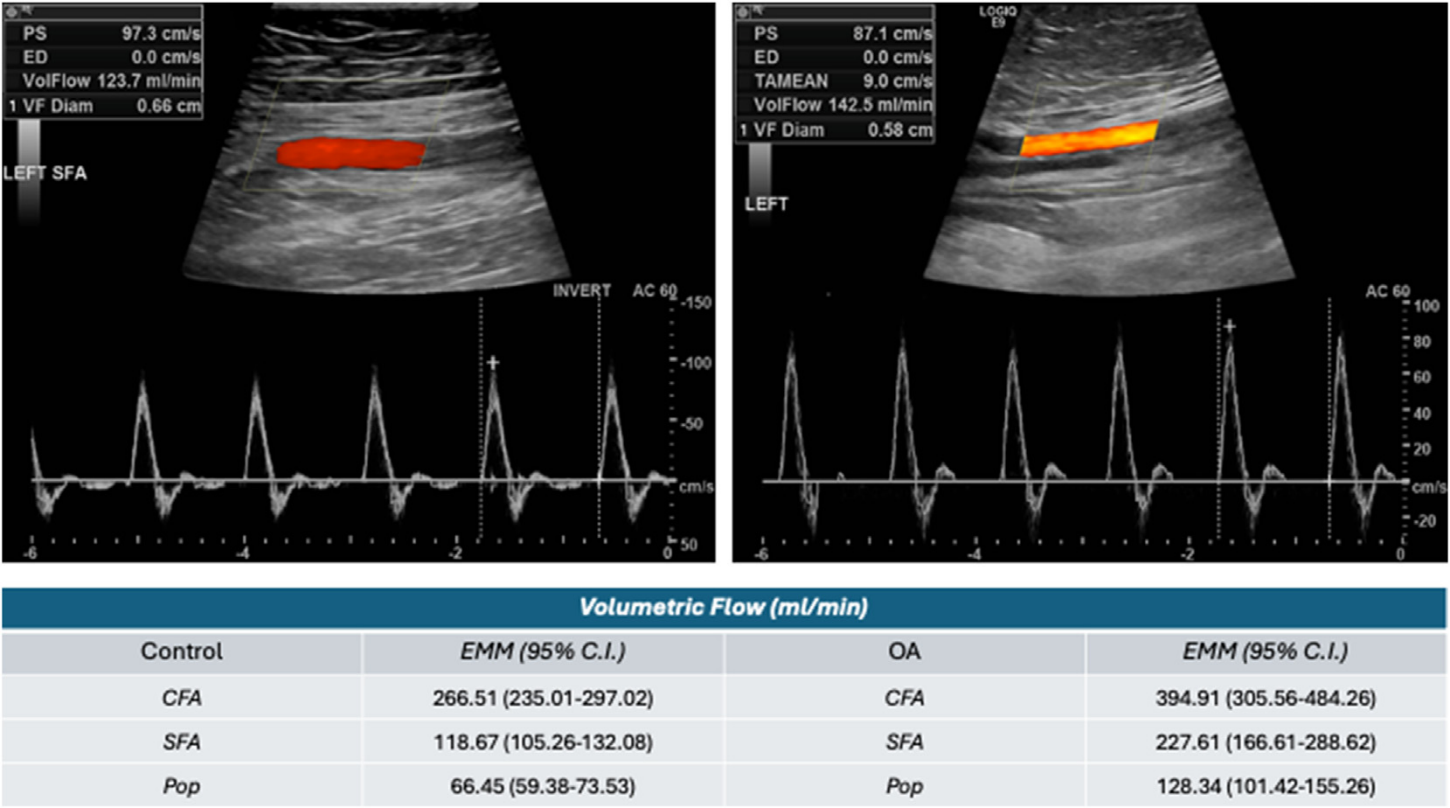
**Limb arterial volumetric flow sonographic imaging captured using the Logiq9 system**. Laminar flow was observed in all major vessels studied. Volumetric flow was significantly greater in all three vessels of OA knees than in the vessels of control knees (CFA *P* ​< ​0.001; SFA *P* ​< ​0.001; Pop *P* ​< ​0.001). EMM ​= ​estimated marginal mean.

Limb arterial blood flow was measured by ultrasound as volumetric flow in ml/min (Fig. 3). The pulse wave velocity (PWV) is defined as the velocity at which the pulse pressure waves, generated by the systolic contraction, propagate in the arteries. It reflects vessel wall material properties, wall thickness, and global structural metrics. Local limb PWV was calculated in the CFA, SFA, and popliteal arteries in duplicate by two separate observers with the Bramwell-Hill equation based upon change in systolic-diastolic cross-sectional luminal area measurements and blood pressure [30]. For the estimation of local limb PWVs, the calculated measurements have been shown to be more precise than measured values based on time and distance [31]. Regional PWV was measured from carotid to femoral arteries as distance/time with time measured by an EKG-gated R wave peak to the foot of the pulse wave upstroke and expressed as m/sec [26,30]. Distances from carotid to femoral measurements were estimated from acromion to femoral pulse. The study was designed with the goal of comparing vascular mechanics as described with the expectation that the OA knees would have pathology reflective of arterial disease.

### 2.3 Statistical analyses

Based upon a previous pilot study investigating volumetric flow and IMT, cohort sizes of 25 knees were estimated to be adequate to detect the effect sizes observed with α ​= ​0.05 and β ​= ​0.10. Sample size was estimated with intent to detect a 1.2 standard deviation difference between OA and control patients while accommodating the Bonferroni adjusted per-comparison alpha (*P* ​< ​0.0002) necessary to maintain an overall alpha of 0.05 across the hypotheses we tested. This effect size was chosen based upon a previous pilot study investigating volumetric flow and IMT. A Bonferroni adjustment was chosen for the purposes of power analysis because it is highly conservative and the Holm test, which was used in all analyses, is based on the empirical *P*-value attained at the time of data analysis which was unavailable. Given these parameters, we estimated that a sample size of 25 knees at the time of analysis would maintain a power of approximately 82.6%.

Generalized estimating equations (GEE) with a main effect for study condition were estimated to compare the study outcome variables between the OA and control conditions. GEE were chosen as the analytic method as they allow appropriate handling of data structures associated with repeated measurements from specimens as seen here with the use of bilateral knees. Classical sandwich estimators were used to protect against possible model misspecification. Model residuals were examined to ensure that all model assumptions were met. Pairwise comparisons between the study conditions were conducted within the model via orthogonal contrasts. The Holm test was used to correct for multiple comparisons and to maintain a 2-tailed familywise alpha at 0.05. All analyses were conducted in SAS version 9.4 (SAS Institute Inc., Cary, NC). A *P*-value <0.05 was used to determine statistical significance, a measure that was interpreted as being clinically important. Results are presented as estimated marginal means and 95% confidence intervals.

## 3 Results

The control cohort consisted of 14 male and 44 female knees with a mean age of 68 years (range, 55 to 77). The OA cohort consisted of 11 male and 24 female knees (approximately 70% female, consistent with the prevalence of females with OA) with a mean age of 68 years (range, ​57 to 80). Ages did not play a role in influencing results. Biological sex did not moderate the differences between control and OA for any of the study outcome variables (*P* ​> ​0.10). For the normal group, mean arterial pressure was 134 ​mmHg (95% CI: 131–138); for the OA group, the mean was 132 ​mmHg (95% CI: 128–138). In the normal cohort, the mean medial joint space width was 4.7 ​mm (95% CI: 4.5–4.9); in the OA cohort, the mean affected joint space width was 1.1 ​mm (95% CI: 0.6–1.6) [*P* ​< ​0.001]. Arterial wall structure and dynamics with the cardiac cycle are presented in Table 1. Knees with OA were found to have arterial walls that were stiffer (less compliant and distensible) than arterial walls in normal knees. Arthritic knees exhibited lesser change in systolic-diastolic luminal area (CFA: *P* ​< ​0.0001, SFA: *P* ​< ​0.02, Pop: *P* ​< ​0.0001), consistent with increased stiffness. Vessel wall IMT was greater in arthritic knees (CFA, SFA, Pop: *P* ​< ​0.0001). The incremental Young's modulus was significantly greater in all three arteries in subjects with knee OA compared to normal knees (CFA, SFA, Pop: *P* ​< ​0.001). These data suggested material changes in the arterial walls that were observed as greater stiffness in the arteries of arthritic knees than in the arteries of normal knees.

**Table 1:** Structural and material properties and flow characteristics of three vessels in the asymptomatic control group (n ​= ​58) and the symptomatic OA group (n ​= ​35).

Variable	Control EMM (95% CI)	OA EMM (95% CI)	*P*-value
Age	68.52 (66.35–70.69)	69.17 (66.31–72.03)	0.72

**Flow characteristics**			

**Volumetric Flow(ml/min)**			
CFA	266.51 (235.01–297.02)	394.91 (305.56–484.26)	0.009
SFA	118.67 (105.26–132.08)	227.61 (166.61–288.62)	0.0009
Pop	66.45 (59.38–73.53)	128.34 (101.42–155.26)	<0.0001
**Pulse wave velocity (m/s)**			
CFA	8.02 (7.40–8.64)	10.00 (9.04–10.95)	0.001
SFA	7.12 (6.56–7.67)	8.87 (8.05–9.70)	0.0008
Pop	7.08 (6.44–7.71)	8.32 (7.55–9.09)	0.02

**Structural characteristics**			

**Intima-media thickness (mm)**			
CFA	0.92 (0.86–0.99)	1.17 (1.11–1.22)	<0.0001
SFA	0.79 (0.74–0.84)	1.07 (0.99–1.14)	<0.0001
Pop	0.83 (0.76–0.89)	1.15 (1.09–1.21)	<0.0001
**Luminal area change (cm**^**2**^**)**			
CFA ΔArea	0.069 (0.058–0.082)	0.032 (0.027–0.037)	<0.0001
SFA ΔArea	0.040 (0.034–0.046)	0.030 (0.024–0.037)	0.02
Pop ΔArea	0.047 (0.039–0.056)	0.017 (0.014–0.020)	<0.0001
**Incremental Young's modulus (MPa)**			
CFA	0.95 (0.79–1.10)	1.73 (1.40–2.06)	<0.0001
SFA	0.92 (0.78–1.06)	1.71 (1.41–2.02)	<0.0001
Pop	0.91 (0.74–1.07)	1.60 (1.29–1.91)	0.0002
**Distensibility (10**^**−**^**^3^/mmHg)**			
CFA	1.35 (1.16–1.54)	0.71 (0.60–0.82)	<0.0001
SFA	1.41 (1.17–1.65)	0.78 (0.60–0.96)	<0.0001
Pop	1.49 (1.22–1.76)	0.78 (0.63–0.92)	<0.0001

EMM ​= ​estimated marginal mean.

Greater arterial stiffness was also reflected in increased blood flow. Volumetric flow was greater in arthritic knees whose arteries were stiffer compared to normal knees which were less stiff (Fig. 3). Local PWVs were calculated for the CFA, SFA, and popliteal arteries with the Bramwell Hill equation and are displayed in Table 1. Local PWV for the OA group was greater in all 3 vessels than in the normal control group. Regional carotid to femoral PWV (cfPWV) was measured using the EKG-gated method of R wave to pulse wave foot [32]. The regional cfPWV reflected clinical meaningfulness with the OA cohort having faster flow than normal controls, indicative of early vascular alterations that may become clinically relevant with time but showed only a trend to statistical significance. The OA group had a mean cfPWV of 8.78 ​m/s (95% CI: 7.83–9.66); the control group exhibited a mean cfPWV of 7.80 ​m/s (95% CI: 7.07–8.46) [*P* ​= ​0.07].

## 4 Discussion

The data presented here demonstrate that patients with OA of the knee have greater arterial wall stiffness by measures of decreased compliance and distensibility, thicker arterial walls (IMT), greater incremental Young's modulus, and faster volumetric blood flow and pulse wave velocity than patients with normal knees. The descriptions of arterial stiffness, volumetric flow, PWV, and other metrics of wall structure and functional flow are closely related physiologically and mathematically, and their abnormalities are physiologically consistent and mutually supportive [30]. These data present, for the first time, a broad assessment of the relationships of stiffer arterial wall biomechanics and blood flow abnormalities comparing subjects with and without OA. The data is supported by studies of similar but isolated observations and clarifies a study in OAfemales with varying and non-physiologic blood flow velocities and IMTthickness [11,13,14].

The measurement of PWV provides complementary information about the arterial elastic properties and stiffness. PWV can be measured regionally, typically carotid-femoral, or locally in peripheral arteries. While regional PWV measurements correlate with systemic cardiovascular diseases, local PWV measurements are more useful in the analysis of arterial wall biomechanical properties [33]. The large heterogeneity of the structure of arterial walls at different sites constitutes an important limitation of regional PWV measurement [34]. Furthermore, local PWV on an arterial segment avoids coarse approximations of the distance between the test points, constituting an important advantage in PWV assessment. A local PWV measurement technique is hence preferred [26]. Higher PWV is reflective of higher arterial stiffness as well as lower vessel distensibility and compliance [35]. Because PWV is a physiological reflection of arterial stiffness, it is an important parameter for risk stratification of vascular disease. Increased arterial stiffness, vascular wall thickness, and incremental Young's modulus are reflected in increased parameters of blood flow and are well recognized hallmarks of early PVD. Their presence in knee OA is consistent with the clinical and phenotypic similarities of patients with OA and PVD and suggest the possibility of shared pathophysiology. These data present an association of increased arterial stiffness and accelerated blood flow in patients with knee OA that can serve as a physiologic platform for mechanistic studies of the apparent association between the common physiologic features of OA and PVD.

How to interpret this data in the context of OA? Clinical, and now physiological, associations between vascular pathology and OA are well established. Mechanistic relationships are less secure and causal relationships have not yet been established. A number of interesting hypotheses linking vascular disease and OA have been put forward based upon the hallmarks of vascular disease seen in OA and the commonality of cytokine expression. One concept considers oxidative stress and low-grade inflammation, both of which are seen in OA and vascular disease [14,36]. Another suggestion is that endothelial cytokines affect angiogenesis in a parallel fashion both in the synovium, for example by inducing changes in levels of MMP1 and VEGF, and even by crossing the subchondral bone plate, introducing osteoblastic and angiogenic cytokines to articular cartilage [[37], [38], [39], [40]]. Yet another proposal is that OA and vascular disease are part of the metabolic syndrome [41]. These conceptualizations depend upon the mutual hyperproduction of signaling and degradative cytokines and growth factors seen under physical stress in arteries (i.e. wall stiffness and increased flow) and under intraosseous hypertension and hypoxia in bone.

Endothelial cells are exquisitely sensitive to alterations in blood flow dynamics and vessel wall stiffness and in response to perturbations in flow and decreased wall compliance, alter their output of many cytokines including among them, notably, nitric oxide, PDGF, TGF-β, IL-1, IL-6, TNFα, and MMP-9 [[42], [43], [44], [45]]. Their dysregulation expresses a myriad of complex, multifunctional, and interactive effects on constituent tissues of joints including matrix breakdown, bone remodeling, and bone neoangiogenesis [46]. The literature on the multifunctionality of cytokine activation in OA of the knee is immense and well beyond the scope of this presentation. However, the major point to be made is that the endothelium, in response to arterial stiffness and associated flow abnormalities, can synthesize a variety of cytokines that have major effects on OA bone and cartilage [43,47,48]. One cytokine of special interest is endothelin. It has been suggested that endothelin may play pathophysiologic roles in subchondral bone remodeling, neovascularization, cartilage breakdown, and synovial capsular inflammation cartilage matrix in OA [[49], [50], [51], [52]]. Endothelin is an endothelial-derived peptide that is released by arterial endothelium in response to arterial wall pathology and aberrant flow of the type demonstrated in this study. Endothelin has profound vasoconstrictive effects on both arterial and bone vasculature and has been of interest as a regulator of bone blood flow for over 30 years [53]. Concentrations of endothelin have been observed to be increased in the plasma of patients with both OA and atherosclerosis, raising the possibility of participation of endothelin in the pathophysiologic processes of both diseases as they relate to blood flow [52,54]. Bone circulation, particularly of the venous vasculature, is exquisitely sensitive to vasoconstriction and endothelin in nanogram doses has been shown to produce venous outflow obstruction and intraosseous hypertension within 10 ​s of intra-arterial infusion, maximizing at 40 ​s [53]. Endothelin has potent vasoconstrictive effects in bone while hypoxia, in turn, has been shown to stimulate endothelin release [55]. While the etiology of venous outflow obstruction in OA bone is unknown, given the sensitivity of bone venous vasculature to vasoconstriction, a plausible hypothesis is that endothelin and/or other cytokines may contribute to the increased venous outflow obstruction and intraosseous hypertension [51,56]. This would begin to provide linkages between vascular-derived cytokines and OA pathology.

The present study has several limitations. All measurements were made non-invasively and should be regarded as estimates of physiology, although clinically relevant. No causation can be inferred but the strength of associations is supported by complementary studies and internally consistent physiological measurements. Local calculations of PWV in lower extremity arteries were quite consistent but regional measurements of aortic PWV encountered recognized limitations of regional carotid femoral PWV determination. Distance measurements between measured flow points were approximations using an externally placed measuring tape. Examination of the arterial pulse waves, and the time from R wave to pulse wave, suggested that those measurements were compromised by up to an 8% variation in some of the time measurements, especially the R wave to the femoral artery flow curve. There is also a significant impedance mismatch between the aorta and the peripheral arteries. For these reasons, while clinically useful, regional PWV is most useful for central rather than limb PWV and can be imprecise for research or physiological use. These observations are in keeping with other reports of measured regional PWV which describe nonaxial flow patterns in the carotid artery resulting in over- or underestimation of blood flow velocity [26]. Although this measurement is clinically associated with systemic cardiovascular disease, from the research perspective, the lack of precision of measurement of carotid-femoral distance, and imprecision of measuring R wave to flow foot diminished the value of the measurement [22]. On the other hand, local PWV measurements provide information of the biomechanical and structural properties of local arterial segments such as those seen in the lower extremity and can more precisely identify local arterial wall stiffness [35,57].

The internally consistent findings in the present study indicate a strong relationship between arterial stiffness and blood flow abnormalities in OA patients compared to patients without OA. These findings are supported by similar studies in different types of OA. The study also presents thought-provoking data, relating arterial stiffness and associated blood flow changes in OA patients, alterations of flow that have been shown to contribute to endothelial dysfunction and cytokine dysregulation [44]. This theory provides potential mechanisms for both vascular and joint disease although much further exploration is needed to determine causality at the biomolecular level. The similarities in phenotype, high prevalence of arterial and cardiovascular disease in OA, and the identified arterial structural and functional anomalies imply a possible relationship. It remains to be shown whether OA and cardiovascular disease are causally related or whether they simply share risk factors, comorbidities, and pathophysiology. It is hoped that the consistency of data in this study will serve as a robust platform for mechanistic studies that may exploit these mutually reinforcing physiological observations.

## Author contributions

Conceptualization, MY, GK, TC and RKA. Investigation, JO, MY, TC and RKA. Data acquisition, JO. Statistical analysis, JO, JM, and MY. Data interpretation, JO, MY, JM, and RKA. Writing—original draft, JO and RKA. Writing—review and editing, JO, MY, JM, GK, DJ and RKA. Project administration, JO and RKA. Funding acquisition, RKA. All authors contributed to editorial changes in the manuscript. All authors read and approved the final manuscript. All authors participated sufficiently in the work and agreed to be accountable for all aspects of the work.

## Funding

This research was funded by a grant from the Miriam Hospital.

## Declaration of competing interest

All authors declare no conflicts of interest.
